# Supplementary material for: Exploring cytokine dynamics in tuberculosis: A comparative analysis of patients and controls with insights from three-week antituberculosis intervention
Source: PLoS One. 2024 Aug 29;19(8):e0305158. doi: 10.1371/journal.pone.0305158 (PMC11361567; doi:10.1371/journal.pone.0305158)
Supplement: S1 Table — (DOCX) [file pone.0305158.s001.docx]

**S1 Table:** **List of included parameters and their definition**

| **Cytokine names** | **Definition** |
| --- | --- |
| Basic FGF | basic fibroblast growth factor |
| CTACK | cutaneous T cell-attracting chemokine |
| Eotaxin | eotaxin |
| G-CSF | granulocyte colony stimulating factor |
| GM-CSF | granulocyte macrophage colony-stimulating factor |
| GRO-α | growth-regulated oncogene-alpha |
| HGF | hepatocyte growth factor |
| IFN-α2 | interferon alpha-2 |
| IFN-γ | interferon-gamma |
| IL-1α | interleukin-1 alpha |
| IL-1β | interleukin-1 beta |
| IL-1ra | interleukin-1 receptor antagonist |
| IL-2 | interleukin-2 |
| IL-2Rα | interleukin-2 receptor alpha |
| IL-3 | interleukin-3 |
| IL-4 | interleukin-4 |
| IL-5 | interleukin-5 |
| IL-6 | interleukin-6 |
| IL-7 | interleukin-7 |
| IL-8 | interleukin-8 |
| IL-9 | interleukin-9 |
| IL-10 | interleukin-10 |
| IL-12 p70 | interleukin-12 (p70) |
| IL-12 p40 | interleukin-12 (p40) |
| IL-13 | interleukin-13 |
| IL-15 | interleukin-15 |
| IL-16 | interleukin-16 |
| IL-17 | interleukin-17 |
| IL-18 | interleukin-18 |
| IP-10 | interferon-gamma inducible protein-10 |
| LIF | leukemia inhibitory factor |
| MCP-1 (MCAF) | monocyte chemoattractant protein-1 |
| MCP-3 | monocyte chemoattractant protein-3 |
| M-CSF | macrophage colony stimulating factor |
| MIF | macrophage migration inhibitory factor |
| MIG | monokine induced by gamma |
| MIP-1α | macrophage inflammatory protein-1 alpha |
| MIP-1β | macrophage inflammatory protein-1 beta |
| β-NGF | nerve growth factor-beta |
| PDGF-BB | platelet-derived growth factor-BB |
| RANTES | regulated on activation, normal T cell expressed and secreted |
| SCF | stem cell factor |
| SCGF- β | stem cell growth factor-beta |
| SDF-1α | stromal cell-derived factor-1 alpha |
| TNF-α | tumor necrosis factor-alpha |
| TNF-β | tumor necrosis factor-beta |
| TRAIL | tumor necrosis factor-related apoptosis-inducing ligand |
| VEGF | vascular endothelial growth factor |
