# Supplementary material for: Exploring cytokine dynamics in tuberculosis: A comparative analysis of patients and controls with insights from three-week antituberculosis intervention
Source: PLoS One. 2024 Aug 29;19(8):e0305158. doi: 10.1371/journal.pone.0305158 (PMC11361567; doi:10.1371/journal.pone.0305158)
Supplement: S2 Table — (DOCX) [file pone.0305158.s002.docx]

**S2 Table: Summary of comorbidities of included patients**

| **Patient Nr.** | **Age** | **Obesity** | **Malnutrition** | **Smoking** | **Alcohol** | **Other diseases potentially influencing cytokine levels** |
| --- | --- | --- | --- | --- | --- | --- |
| 1 | 42 | slightly overweight | no | yes | no | intramural leiomyoma of the uterus, bilateral retinal necrosis |
| 2 | 47 | no | no | yes | NA | trichiuriosis |
| 3 | 63 | no | severe malnutrition | yes | yes | extensive tumor in the otorhinolaryng, onychomycosis, chronic obstructive pulmonary disease, bronchiectasis, centrilobular emphysema |
| 4 | 43 | no | no | yes | occasionally |  |
| 5 | 47 | no | yes | yes | no | clostridial enterocolitis |
| 6 | 47 | no | no | yes | occasionally | pneumothorax |
| 7 | 49 | yes | no | yes | occasionally | diabetes mellitus 2, hyperlipidaemia, pulmonary sarcoidosis |
| 8 | 52 | no | severe malnutrition | yes | NA | malignant tumor of the tonsils, secondary tumor of the lymph nodes of the head, face and neck, secondary malignant lung tumor, Covid infection |
| 9 | 28 | no | no | yes | NA |  |
| 10 | 60 | slightly overweight | no | yes | yes | chronic ethanol-induced hepatopathy, chronic obstructive pulmonary disease, Covid infection |
| 11 | 23 | no | severe malnutrition | yes | NA | left kidney cyst, ascariasis of the small intestine, scabies, hepatomegaly |
| 12 | 24 | no | severe malnutrition | yes | yes |  |
| 13 | 55 | no | no | yes | yes | diabetes mellitus 2, hyperlipoproteinemia |
| 14 | 37 | no | severe malnutrition | yes | no | fluidothorax, exudative non-biliary pancreatitis, hepatitis |
| 15 | 36 | no | yes | yes | yes | enterocolitis - Clostridium difficile, chronic rhinitis, tooth decay, scabies |
| 16 | 68 | no | no | no | yes | rhabdomyolysis, renal insufficiency |
| 17 | 52 | no | no | yes | yes | ethanol-induced hepatopathy, fluidopneumothorax |
| 18 | 43 | no | no | yes | yes | hepatopathy, stomach ulcer disease |
| 19 | 44 | no | severe malnutrition | yes | yes | hypoxemic respiratory insufficiency with hypercapnia |
| 20 | 58 | no | no | yes | yes | hyperuricemia, chronic ulcer of the foreleg, MRSA in the wound, diabetes mellitus 2 |
