# Supplementary material for: Exploring cytokine dynamics in tuberculosis: A comparative analysis of patients and controls with insights from three-week antituberculosis intervention
Source: PLoS One. 2024 Aug 29;19(8):e0305158. doi: 10.1371/journal.pone.0305158 (PMC11361567; doi:10.1371/journal.pone.0305158)
Supplement: Attachment — Submitted filename: Response to reviewers.docx [file pone.0305158.s004.docx]

RESPONSE TO REVIEWERS

Thank you very much for the initial review of our study and for your suggestions for further improvement. Here, we react to all your comments and the relevant revisions have been made also in the manuscript. All authors approved the revisions and the latest version of the manuscript.

**This is a good study. 
There is novelty in this study. 
I give some comments for improvement before proceed to the reviewers. 
- Please improve the quality of the figure.**

We have improved the quality of the figures 3 and 4 and uploaded the newer versions. Thank you for this suggestion.

**- Please give detail the design of your study.**

We have added the design of this study in the Methods section as well as inclusion and exclusion criteria. The following sentences have been edited:

line 72 - *In this prospective cohort study …*

line 74-76 - *The inclusion criteria were as following: age ≥ 18 years and diagnosis of active form of pulmonary TB confirmed by bacteriologic culture. Patients with latent or extrapulmonary forms and recurrent TB were excluded.*

**- Please begin the discussion with the main findings of your study.**

Thank you for this comment. We began the discussion with the following sentences:

*Our study showed elevated levels of 31 cytokines in TB patients compared to healthy controls. Three-week anti-TB treatment led to decreased levels of 9 of them that could potentially reflect treatment efficacy.* (line 163-164)

**- Please elaborate more the limitations of your study.**

We have edited the limitations of the study as follows:

line 259 - *This study has several limitations.*

line 262-263 - *Longer follow-up would provide a more comprehensive understanding of how cytokine levels evolve throughout the treatment process.*

line 265-267 - *Moreover, we are aware that such small sample size cannot adequately represent the diverse population of TB patients considering also their various comorbidities.*

**- The discussion section is too long.**

The discussion section was shortened. We updated the references accordingly.

**- The conclusion should be concise and clear.**

We reworded the conclusion section making it more concise and highlighting the main findings:

*In conclusion, our study sheds light on cytokine dynamics during the initial three weeks of anti-TB treatment and identifies elevated cytokines in active pulmonary TB compared to healthy controls. We observed significant reduction in levels of 9 cytokines, including TNF-α, IL-12 p40, IFN-α2, and LIF, after three weeks of therapy, highlighting their potential of early treatment monitoring. Additionally, our correlation analysis suggests a potential association between cytokine levels and BMI, emphasizing the importance of considering host factors in TB pathogenesis and treatment response. Such analysis could be further performed in paediatric patients to compare the infection dynamics and establish the cytokine profiling for TB diagnosis and treatment response in younger population.* (line 270-277)
