# Supplementary material for: Exploring cytokine dynamics in tuberculosis: A comparative analysis of patients and controls with insights from three-week antituberculosis intervention
Source: PLoS One. 2024 Aug 29;19(8):e0305158. doi: 10.1371/journal.pone.0305158 (PMC11361567; doi:10.1371/journal.pone.0305158)
Supplement: Attachment — Submitted filename: Response to reviewers.docx [file pone.0305158.s005.docx]

Dear editor and dear reviewers,

First of all, thank you very much for the additional comments and suggestions that contributed to the higher quality of the article. We have modified the manuscript according to your comments and every each change is discussed point by point below in the document.

**Response to Editor and Reviewer #1**

**Reviewer #1: The study addresses a critical gap in TB treatment by exploring cytokine levels as potential biomarkers for monitoring the disease's progression and response to treatment. The results are quite interesting; however, the paper has several notable deficiencies that detract from its overall impact:**

**The study's small sample size and short observation period significantly undermine the strength and applicability of the findings. With only 20 TB patients studied over three weeks, it is difficult to generalize these results to the broader TB patient population. The conclusions drawn from such a limited dataset risk being premature, particularly concerning the efficacy and durability of treatment responses:**

Dear Editor and Reviewer, thank you very much for this helpful comment. We are aware of the small cohort of patients, so we have included this information in the study limitations. However, this is due to several factors:

1. Slovakia is among the countries with a low incidence of tuberculosis (an average of 130 cases pulmonary tuberculosis per year), of which approximately 40% of cases are diagnosed in the pediatric population, which was not included in the study.
2. The study was conducted in collaboration with the facility with the highest number of hospitalized patients in Slovakia: National Institute for Tuberculosis, Lung Diseases and Thoracic Surgery, Vyšné Hágy, Slovak Republic. However, some patients are also hospitalized in regional hospitals, which has contributed to a lower cohort of patients, as we do not actively cooperate with these facilities (many of them are private).
3. Patients with drug-resistant tuberculosis were excluded due to prolonged treatment regimens and the expected slower reflection of the effectiveness of therapy on the cytokine profile.
4. Patients with extrapulmonary TB and HIV were also excluded.

Regarding the observation period, following the improvement of the condition (in case of drug-susceptible tuberculosis), patients are often discharged for outpatient treatment three weeks after the initiation of therapy. To ensure uniform sampling times and to collect samples from as many patients as possible, we opted to sample on the day treatment commenced and after 7 and 21 days.

**While the study includes a control group, further details about its selection and characteristics could help strengthen the comparative analysis. It is crucial to ensure that the control group closely matches the patient's demographic status. I couldn't find such information in the text.**

Thank you for this comment regarding the control group. The individuals included in this group were carefully selected to be age-matched with the patients and the equal number of females and males was chosen to make this group representative. Controls as well as patients in this study were Caucasians from Slovakia. Controls did not suffer from any infectious and inflammatory diseases, nor from any oncology diagnosis.

**The study does not consider the complex interactions between different cytokines or external factors that might influence cytokine levels, such as concurrent infections or underlying chronic conditions. This oversimplification can lead to misleading conclusions about the role and significance of specific cytokines in TB treatment response.**

Considering the potential interactions between cytokines and external factors, in such a small group with variable comorbidities we did not further divide them into subgroups according to their underlying conditions as the outcomes would not be sufficiently representative. We prepared a table summarizing all comorbidities for each patient (see Supplementary Table 2.). We considered only the effects of various treatment regimens on the levels of cytokines, which has been mentioned in the study. Also, due to the pleiotropic nature of cytokines and their complex interactions, correlation analysis would likely produce numerous results without yielding any significant conclusions.

**The study finds a correlation between cytokine levels and BMI, suggesting that nutritional and metabolic factors affect the immune response to TB. Expanding this research to include more comprehensive metabolic profiling could uncover additional insights into how these factors influence treatment outcomes**

Thank you for this comment. We agree that inclusion of comprehensive metabolic profiling could bring new important insights into the pathophysiology and treatment impact of pulmonary TB. In the future, we want to expand the patient cohort to include pediatric patients and metabolomic analyses.

**While the study focuses on cytokines as markers of treatment efficacy, exploring their role in predicting treatment failure or complications could also be valuable. Additionally, examining cytokine profiles in conjunction with other biomarkers, such as genetic or molecular markers, might provide a more holistic view of the disease and its management.**

This is actually our plan for future studies. We have started with the analysis of genetic (SNPs) and epigenetic markers (miRNAs) related to the diagnosis of TB and we are also expanding our sample size.

**Reviewer #2: The study has novelty and can be published. It will be a useful addition for further studies and research. Though clinical parameters are of paramount importance, the inflammatory markers may help aid the prognosis at an early stage.**

We would like to thank Reviewer 2 for the time dedicated to the review of our manuscript and for the positive comments.
